# Single-Molecule Mechanical Analysis of Strand Invasion in Human Telomere DNA

**DOI:** 10.1021/acs.biochem.1c00448

**Published:** 2022-07-19

**Authors:** Terren R. Chang, Xi Long, Shankar Shastry, Joseph W. Parks, Michael D. Stone

**Affiliations:** † Department of Chemistry and Biochemistry, University of California, Santa Cruz, 1156 High St, Santa Cruz, California 95064, United States; ‡ 10X Genomics, 6230 Stoneridge Mall Rd, Pleasanton, California 94588, United States; § Invitae, 1400 16th St, San Francisco, California 94103, United States

## Abstract

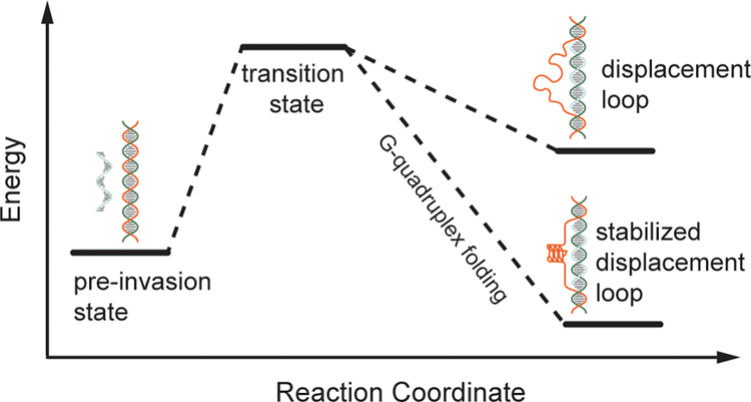

Telomeres are essential chromosome end capping structures that safeguard the genome from dangerous DNA processing events. DNA strand invasion occurs during vital transactions at telomeres, including telomere length maintenance by the alternative lengthening of telomeres (ALT) pathway. During telomeric strand invasion, a single-stranded guanine-rich (G-rich) DNA invades at a complementary duplex telomere repeat sequence, forming a displacement loop (D-loop) in which the displaced DNA consists of the same G-rich sequence as the invading single-stranded DNA. Single-stranded G-rich telomeric DNA readily folds into stable, compact, structures called G-quadruplexes (GQs) in vitro and is anticipated to form within the context of a D-loop; however, evidence supporting this hypothesis is lacking. Here, we report a magnetic tweezers assay that permits the controlled formation of telomeric D-loops (TDLs) within uninterrupted duplex human telomere DNA molecules of physiologically relevant lengths. Our results are consistent with a model wherein the displaced single-stranded DNA of a TDL fold into a GQ. This study provides new insight into telomere structure and establishes a framework for the development of novel therapeutics designed to target GQs at telomeres in cancer cells.

## Introduction

Telomeres safeguard the genome by distinguishing chromosomal termini from sites of DNA lesions that would otherwise elicit an unwanted DNA damage response, resulting in chromosomal fusion, genomic instability, and often apoptosis.^1,2^ The foundation of the telomere structure begins with tandem hexameric guanine-rich (G-rich) repetitive DNA (GGTTAG in humans) ∼2 to 20 kilobases in length^3,4^ and terminates with a ∼50 to 300 nucleotide long single-stranded G-rich 3′ overhang.^5^ Telomeres also act to buffer against the end replication problem, wherein chromosomes gradually shorten with each subsequent round of cell division.^6^ Replication-dependent telomere attrition can compromise the protective function of telomeres as well as lead to a loss of genetic information if left unaddressed.^7^ Therefore, continually dividing cells, including the majority of human cancers, must maintain the telomere length to support an immortal phenotype.^8,9^ A majority of proliferative cell types upregulate the specialized enzyme telomerase, which reverse transcribes telomeric DNA on to chromosomal termini using an RNA template that resides within the integral telomerase RNA subunit.^10−13^ However, many aggressive cancer subtypes employ a telomerase-independent mechanism for telomere maintenance termed alternative lengthening of telomeres (ALT).^14^ In ALT cells, the 3′ single-stranded DNA (ssDNA) overhang of one telomere base pairs with the duplex region of another telomere, in a manner similar to early steps in homology directed repair.^15^ This telomeric strand invasion event forms a displacement loop (D-loop), where the single-stranded G-rich 3′ overhang base pairs with the C-rich strand of the invaded telomere, displacing the G-rich strand.^16−18^ The 3′ overhang can then be extended by a specialized DNA polymerase using the invaded telomere as a template,^19^ followed by the synthesis of the C-rich strand and nucleolytic processing to maintain the 3′ overhang.^20^

Single-stranded G-rich telomeric DNAs readily fold into compact structures called G-quadruplexes (GQs) in vitro, wherein guanine bases form G-quartets via both Watson–Crick and Hoogsteen base-pairing interactions to align in a plane while coordinating a monovalent cation at the center. Multiple G-quartets can in turn stack upon each other to form a GQ.^21^ The stability of GQs is highly dependent on the identity of the monovalent cation, with a rank order of K^+^ > Na^+^ > Li^+^, in terms of the degree of stabilization.^22^ Furthermore, small molecule ligands designed to target GQ structures elicit a phenotype in living cells, suggesting a possible regulatory role for these structures in vivo.^23^ Therefore, much effort has been put forth to identify potential GQ forming sequences in the genome to expand the potential targets for these molecules to be used as therapeutics.^24^ In the current study, we report results from a single-molecule mechanical assay of DNA strand invasion at human telomeres. Using a magnetic tweezers system, uninterrupted duplex telomere DNA molecules greater than seven kilobases can be manipulated in order to impart precise degrees of tension and torque to the system. Strand invasion by single-stranded DNA oligonucleotides in solution can be monitored real time as a change in the overall extension of the telomere DNA duplex target molecule. To our knowledge, this assay is the first to permit direct detection of telomeric D-loops (TDLs) at the single-molecule level. We find that conditions that disfavor GQ folding dramatically alter the properties of TDLs, suggesting a role for GQ folding within these important structures. Finally, this system provides an experimental framework for future single-molecule studies of small molecule drugs and cellular machinery that may bind and alter the GQ structure within a TDL.

## Results

### Single-Molecule Manipulation of Long Human Telomere DNA Molecules

**Figure 1:**
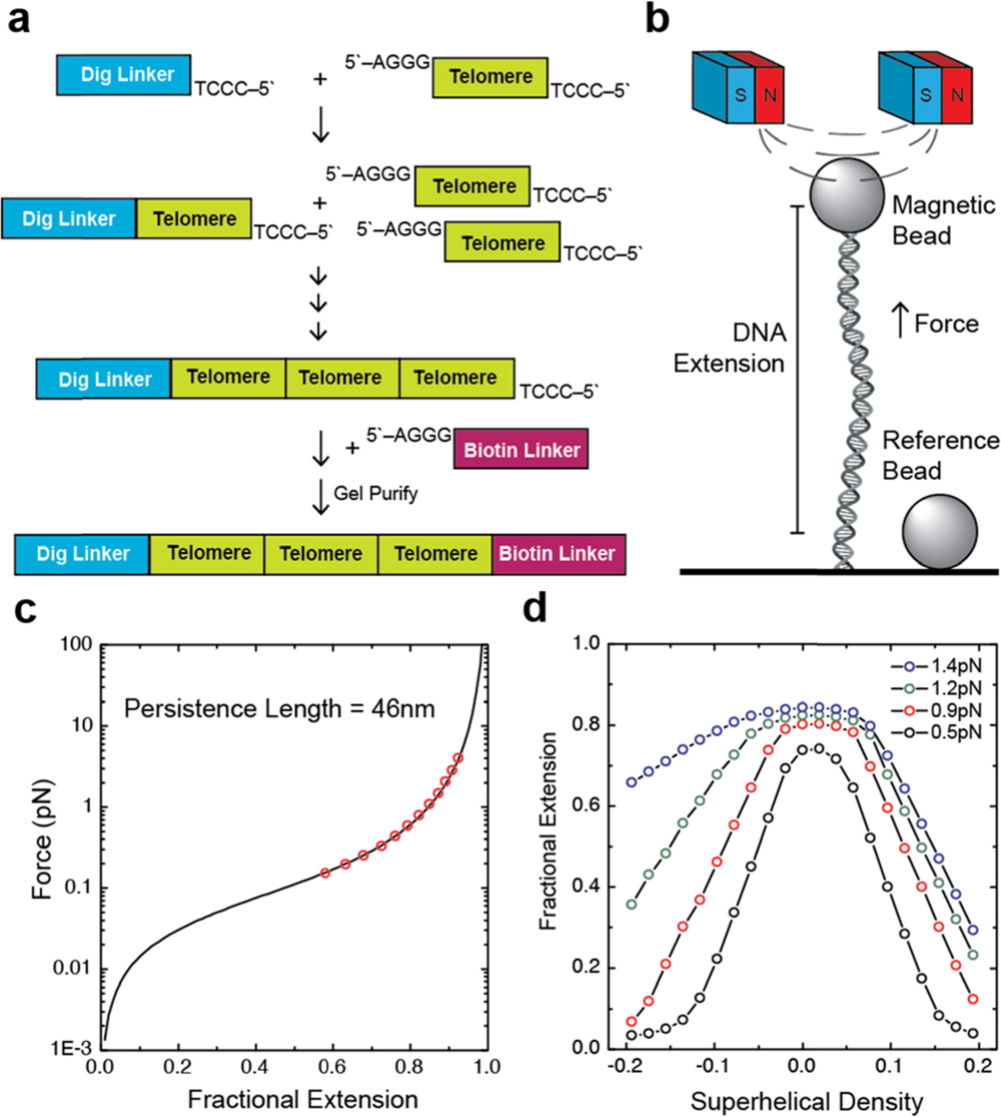
Mechanical properties of long duplex telomeric DNA. (a) Schematic of the construction of telomeric DNA molecules used in this study. (b) Schematic of the magnetic tweezers instrument. Vertical position of the magnets is adjusted to control the force exerted on the tethered DNA molecule. Magnets can also be rotated to apply torque. (c) Force-extension curve of telomeric DNA. Data points are in red with the wormlike chain fit in black. (d) Rotation-extension curves of telomeric DNA at various forces.

The DNA molecules used in the present work consist of greater than seven kilobases of uninterrupted double-stranded telomeric DNA. The telomere DNA molecule is flanked by biotin- or digoxigenin-modified DNA linker fragments used to immobilize the DNA tether between a streptavidin-coated magnetic bead and an anti-digoxigenin-coated glass slide, respectively (Figure 1A, B). To generate these long, uninterrupted, telomere DNA tether molecules for single-molecule analysis in our magnetic tweezers microscope, we perform a controlled DNA concatenation reaction seeded on the digoxigenin linker fragment using a 576 base pair telomere DNA fragment with compatible sticky ends generated by the restriction endonuclease cleavage of the previously reported pRST5 DNA plasmid.^25^ Following multiple rounds of DNA ligation, the molecule is ultimately capped by the ligation of the biotin-modified DNA linker fragment and gel purified to remove unwanted reaction side products and excess handle material (Figures 1A and S1, see Methods for details of DNA molecule construction).

The elasticity of double-stranded DNA is well described using the wormlike chain (WLC) polymer model^26^ and is characterized by a bending persistence length ranging from ∼45 to 50 nanometers (nm), depending upon the ionic strength.^22,27^ To test whether our telomere DNA tethers exhibit similar elastic properties, we performed force-extension analysis. Our results indicate that long double-stranded telomere DNA molecules exhibit canonical DNA elastic properties with an average persistence length of 46 ± 4 nm under the conditions of our experiments (10 mM Tris pH 7.5, 150 mM KC_2_H_3_O_2_, 0.5 mg/mL BSA) (Figure 1C). Next, we analyzed the supercoiling response of our telomere DNA tethers by rotating the magnets held above a molecule of interest, which permits the precise introduction of positive or negative superhelical strain into the system. DNA tether extension data are collected for a variety of superhelical densities, given by the expression σ = Δ*L*_k_/*L*_ko_, where σ is the supercoiling density, Δ*L*_k_ is the change in the DNA linking number (i.e., the integer number of magnet rotations), and *L*_ko_ is the linking number of the DNA molecule in a topologically relaxed state (i.e., the total number of DNA base pairs in the tether divided by the number of base pairs per helical turn of the double helix) (Figure 1D). Interestingly, we find that telomeric DNA more readily denatures in response to the applied negative superhelical strain when compared to a nontelomeric control DNA (Figure S2), consistent with a recently reported study of force-induced denaturation of a nontelomeric GQ forming sequence.^28^

### Real-Time Observation of DNA Strand Invasion in Human Telomere DNA

**Figure 2:**
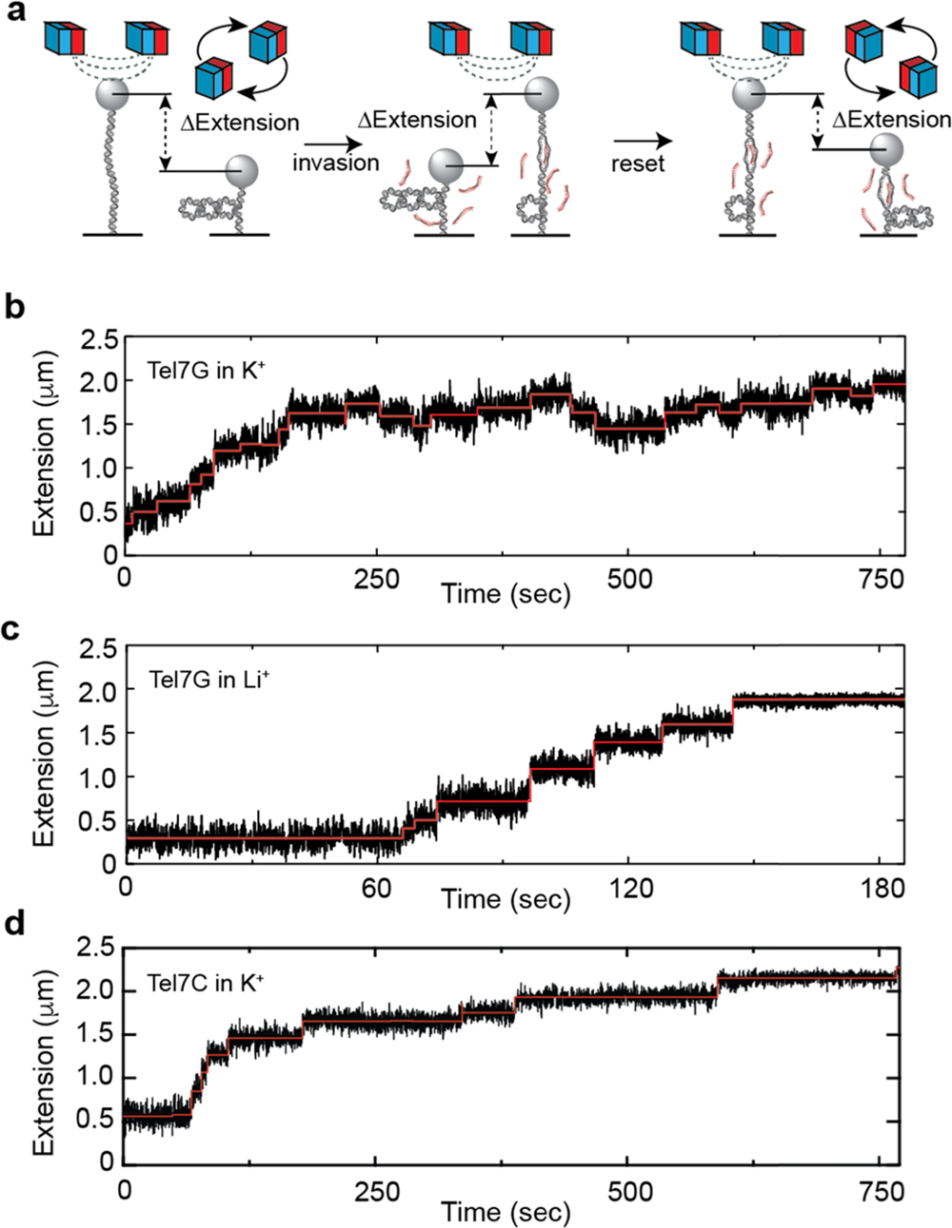
Real-time detection of telomeric D-loop formation by strand invasion. (a) Schematic of the magnetic tweezers experimental setup. Initially, a single telomere DNA molecule is tethered between a glass slide and a magnetic bead, followed by the introduction of negative supercoils. Spontaneous strand invasion events are detected as discrete, stepwise increases in DNA tether extension. The molecule is then positively supercoiled while monitoring the extension to eject the invaded strands. (b) Real-time trajectory of a Tel7G (TTAGGG)_7_ strand invasion experiment in K^+^. Raw DNA extension signal (black) is overlaid with an idealized trace generated by a step-finding algorithm (red). (c) Real-time trajectory of a Tel7G (TTAGGG)_7_ strand invasion experiment in Li^+^. (d) Real-time trajectory of a Tel7C (CCCTAA)_7_ strand invasion experiment in K^+^.

Having characterized the physical properties of the telomere DNA tethers, we next developed a DNA topology-based assay to directly measure telomere DNA strand invasion in real time (Figure 2A). The molecule is initially negatively supercoiled resulting in a decrease in extension. When a stretching force is applied, the negative superhelical density imparts torque on the molecule, which results in transient, local destabilization of the DNA double helix and facilitates strand invasion by a freely diffusing complementary DNA oligonucleotide from solution^29^ (Figure 2A, middle panel). In this assay, the negatively supercoiled telomere DNA tether represents a closed topological system. Therefore, the local DNA unwinding that must occur upon strand invasion induces compensatory positive supercoiling, which in turn cancels some of the preexisting negative supercoiling, resulting in a sudden increase in the DNA tether extension when held at constant force (Figure 2B–D). In this way, many successive strand invasion events on a single telomere DNA molecule are measured in real time.

To initially characterize strand invasion in our system, we monitored the properties of a 42 nucleotide long single-stranded invading DNA molecule composed of seven repeats of the G-rich telomere DNA strand sequence (Tel7G) in the presence of K^+^, the physiologically relevant monovalent cation. We elected to conduct strand invasion experiments at the force set point of 0.9 pico-Newtons (pN), as this amount of tension is sufficient to facilitate strand invasion on tractable timescales but is not enough to stably denature the telomere DNA tether as evidenced by the symmetric rotation-extension curves collected at this stretching force (Figure S2).

In the presence of 10 nM Tel7G, we observe discrete changes in the extension of the telomere DNA tether, with the majority of transitions resulting in an increase in the extension of the system, as expected for stable strand invasion events (Figure 2B). Notably, we also observe reverse transitions to shorter extensions, suggesting that the invading strand may also dissociate or reorganize during the observation time. Importantly, we only observe the discrete transitions in the presence of the complementary invading strand (Figure S3), supporting the interpretation that the cumulative increase in the telomere DNA extension over time is due to the accumulation of invading strands that result in the formation of TDLs.

### G-Rich Telomere DNA Exhibits Complex Dynamics during Strand Invasion

As noted above, the displaced strand upon invasion of the Tel7G oligonucleotide consists of multiple G-rich telomere DNA sequence repeats and therefore is expected to fold into a GQ structure in the presence of K^+^. In order to explore the possible effects of the GQ structure on the strand invasion process, we conducted the same Tel7G invasion experiment in the presence of Li^+^, a condition that is known to destabilize GQ folding (Figure 2C). We noted that the kinetics of invasion is markedly faster at the same force set point of 0.9 pN and the same concentration of the invading strand (compare Figure 2B,C). One possible explanation for this observation is that the target DNA duplex is less energetically stable in the presence of Li^+^ when compared to K^+^, a feature of B-form DNA that, to our knowledge, has not been biophysically characterized. To analyze this possibility, we compared the extension properties of the telomere DNA tethers as a function of superhelical density in both K^+^ and Li^+^. Indeed, we find that the DNA is more readily denatured by applied torques in the presence of Li^+^ (Figure S4), which provides an explanation for the increased rate of Tel7G invasion observed in our experiments. The effect of Li^+^ on the torsional stability of B-form DNA is not telomere-specific, as we observe a similar behavior for a nontelomeric DNA tether studied in K^+^ vs Li^+^ (Figure S4). Interestingly, when invasion was conducted in the presence of Li^+^, we observed a significant decrease in the prevalence of reverse steps (Figure S5), which as described above, may be due to dissociation of invading strand and/or reorganization of the invaded DNA structure. Given the known destabilizing effect of Li^+^ on GQ folding, this effect suggests that GQ formation may contribute to the propensity for reverse steps during telomeric DNA strand invasion.

**Figure 3:**
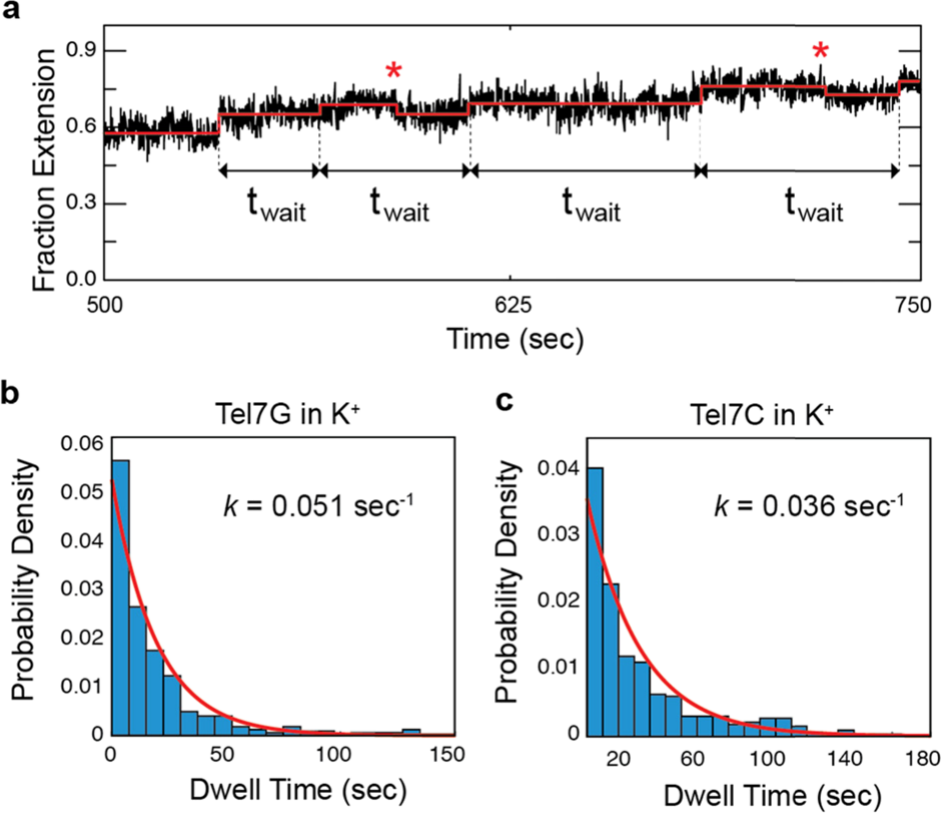
Kinetic analysis telomeric strand invasion. (a) Representative plot depicting the details of kinetic analysis of telomere strand invasion. Extracted dwell times (*t*_wait_) between forward invasion steps are shown, with occasional backward steps indicated by red asterisks. Binned dwell time distributions for strand invasion by Tel7G or Tel7C, with the MEMLET fits to a single exponential function superimposed in red. The extracted rate constants for invasion are shown in panels (b) and (c). The 95% confidence interval determined by bootstrapping for each rate constant is 0.046–0.058 for Tel7G and 0.032–0.040 for Tel7C.

As a further test of this possibility, we next set out to investigate differences in the strand invasion dynamics observed for the complementary C-rich Tel7C oligonucleotide (Figure 2D). Comparison of strand invasion trajectories collected for the Tel7C and Tel7G invading strands in K^+^ reveals an obvious qualitative difference (compare Figure 2B, D), wherein the Tel7C invasion trajectories primarily consist of a stepwise monotonic increase in the observed DNA tether extension with a reduced frequency of reverse steps (Figures 2D and S5). To analyze whether the reverse steps are mechanistically coupled to the forward steps, we analyzed the dwell time distributions for individual invasion events. Kinetic analysis was performed by measuring the waiting times (*t*_wait_) between successive invasion events, irrespective of whether there was an intervening reverse step observed in the trajectory (Figure 3A). Despite the qualitative differences we observe in the invasion trajectories for Tel7G and Tel7C in K^+^, we find both dwell time distributions to be well described by single exponential functions with similar characteristic rate constants for invasion (Figure 3B, C). This result suggests that the forward and reverse steps in the real-time invasion trajectories are independent of each other and demonstrate that the rate constant for strand invasion is comparable for the Tel7G and Tel7C invading strands.

In addition to the potential of the displaced strand in a TDL to form a GQ in the presence of K^+^, it is also possible that GQ formation in the Tel7G invading strand itself may occur and contribute to the increased prevalence of reverse steps. To investigate this possibility, we performed control experiments with a modified G-rich invading oligonucleotide in which the second base of alternating G-triplet sequences was replaced with a 7-deazaguanine-modified base (Tel7dG). Invading oligonucleotides harboring this 7-deazaguanine modification cannot form the requisite Hoogsteen hydrogen bonds required to form a stable GQ structure.^30^ When using Tel7dG, we found that the frequency of reverse steps in the invasion traces was comparable to that observed with the native Tel7G sequence (Figure S6). Thus, the observed dynamics are not dependent upon GQ formation within the invading G-rich strand. Taken together, these results support a model wherein GQ folding in the displaced strand of a TDL at least in part contributes to the increased prevalence of reverse steps observed in Tel7G strand invasion trajectories in K^+^ conditions. However, kinetic analysis suggests that the reverse steps do not significantly impact the rate constant for the invasion and formation of the TDL structure.

### Structural Stability of TDLs Formed by G-Rich Strand Invasion

If the displaced strand within a TDL formed upon G-rich strand invasion folds into a GQ structure, one prediction is that the TDL will be less energetically favored to resolve because the H-bonds that have been disrupted upon strand invasion are compensated by H-bonds within a GQ fold. As noted above, the stable unwinding of the telomere DNA target during strand invasion results in a change in the overall DNA twist (i.e., the number of helical turns per unit length of the DNA molecule). Such changes in DNA twist, if structurally stable, can be directly measured as a shift in the rotation-extension curve to the left when the magnets are rotated back toward the relaxed state of the DNA molecule.^29^ In contrast, if the invading strands dissociate while rewinding the molecule back toward the relaxed state (i.e., TDL resolution), one would expect to observe a rotation-extension curve that overlays the original preinvaded state.

**Figure 4:**
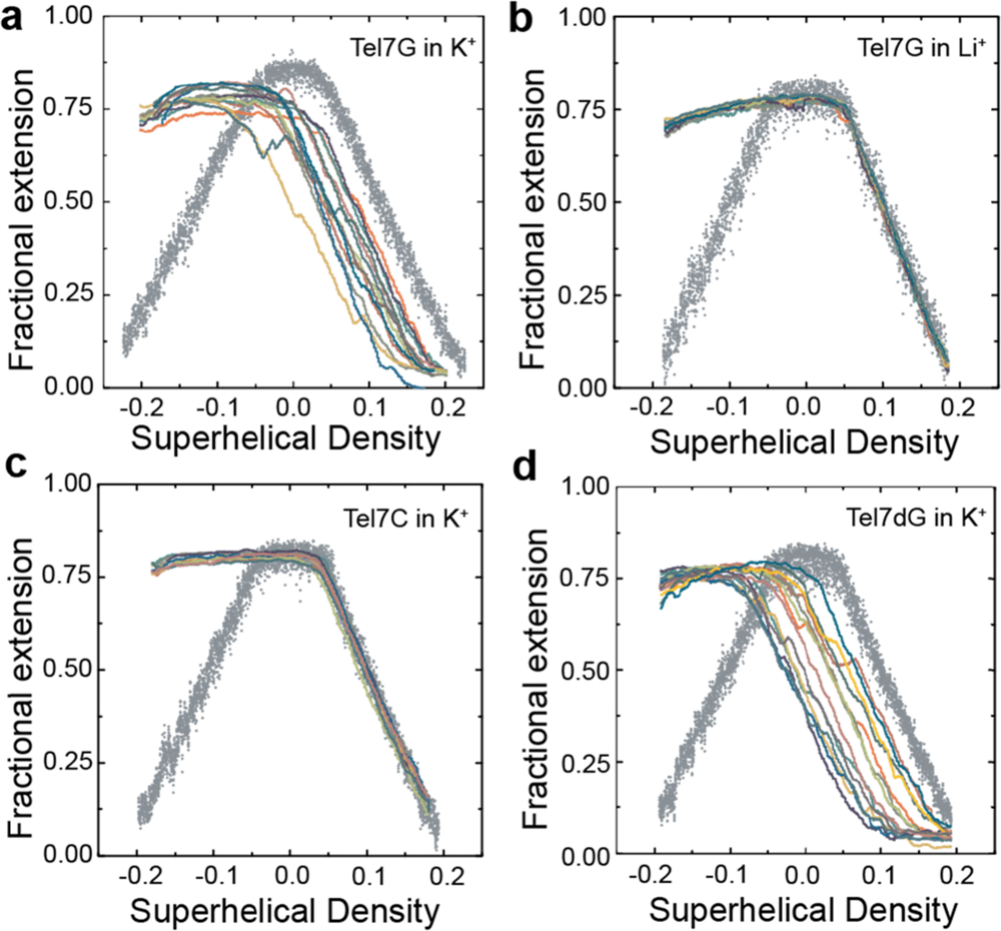
Stable telomeric D-loops result in a shift of the rotation-extension curve. Initial rotation-extension curves in the absence of invading strands are shown in gray dots, while individual replicates (*n* = 14) of rotation-extension data collected following complete strand invasion are shown in colored solid lines (*n* = 14 for all panels). (a) Tel7C in K^+^. (b) Tel7G in K^+^. (c) Tel7G in Li^+^. (d) Tel7dG in K^+^.

After complete invasion of a target telomere DNA tether with the Tel7G oligonucleotide (defined as the DNA tether reaching >70% of its relaxed extension), the magnets were rotated back toward the relaxed state and into the positive superhelical density regime. The overlay of multiple independent rotation-extension curves taken following Tel7G invasion reveals a significant shift in the rotation-extension curve along the *x*-axis (Figure 4A). This hysteresis in the rotation-extension curve on a molecule invaded by the Tel7G strand is indicative of increased structural stability of TDLs when the physiologically relevant G-rich invading strand is used in K^+^. Interestingly, if the same experiment is performed with the Tel7G strand in the presence of Li^+^ rather than K^+^ (Figure 4B), or with the Tel7C strand in K^+^ (Figure 4C), the observed hysteresis in the rotation-extension curve is eliminated. Taken together, these results demonstrate that both a G-rich invading strand and GQ favoring conditions (i.e., K^+^) are necessary for the increased stability of the TDL.

Although our results are consistent with a role for GQ in stabilizing TDL structures formed upon Tel7G invasion, it remained a possibility that the G-rich invading strands in solution participate in intermolecular GQ formation with the displaced strand, rather than formation of an intramolecular GQ within the displaced strand of the TDL. To distinguish between these two possibilities, we again turned to the use of the modified Tel7dG oligonucleotide, which cannot form GQs but preserves Watson–Crick base pairing.^30^ Analysis of DNA tethers following strand invasion by the Tel7dG strand again revealed a leftward shift of the rotation-extension curve, consistent with stable TDL formation (Figure 4D). These data lend further support to the notion that intramolecular GQs formed within the displaced strand of TDLs structurally stabilize the invaded state.

## Discussion

Magnetic tweezers (MT) force spectroscopy is a powerful tool to probe DNA mechanics.^31−33^ MT-based methods have been applied to the study of human telomere DNA in recent years, with a focus on the propensity of this repetitive G-rich sequence (GGTTAG)_*n*_ to fold into G-quadruplex (GQ) structures.^33−35^ Previously published single-molecule spectroscopic analyses of telomere DNA mechanics have largely focused on the structural properties of short single-stranded (ss) model telomere DNA substrates.^33,34,36−41^ In the present work, we use a MT system to interrogate the structural properties of long, uninterrupted duplex telomere DNA molecules of physiologically relevant lengths (>7 kilobases).

MT methods have also previously been used to directly monitor DNA strand invasion in real-time, providing a tool to study the mechanics of this essential DNA transaction that occurs during DNA repair and recombination pathways.^29,42^ Here, we have adopted this approach to study strand invasion at telomere DNA target sites, a process proposed to occur during the formation of telomere-loops (T-loops) as well as during the ALT pathway.^15−18^ By using telomeric ssDNA probes of physiologically relevant lengths introduced to individual duplex telomere DNA molecules held under precisely applied degrees of superhelical strain, we detect real-time strand invasion and the formation TDLs. The ability of applied torque to a telomere DNA target molecule to facilitate strand invasion supports a previous model for the role of the telomere repeat binding factor 2 protein (TRF2), which has been shown to wrap duplex telomere DNA in a chiral fashion, resulting in the application of negative superhelical strain and promoting T-loop formation.^43,44^

Interestingly, we observe complex invasion dynamics when the invasion trajectories are collected in the presence of a G-rich ssDNA oligonucleotide, intended to model the G-rich 3’ ssDNA tail that exists at endogenous telomere ends. The invasion dynamics are characterized by a combination of forward and reverse steps, and these reverse steps are suppressed when performing the same experiments in the presence of Li^+^ or when using the complementary C-rich strand for invasion. It is well established that Li^+^ has a destabilizing effect on GQ folding.^21^ We also provide evidence that TDLs formed upon G-rich strand invasion in the presence of K^+^ are more energetically stable than those when formed in the presence of Li^+^ or with the C-rich strand. Taken together, these results lead to a model wherein the formation of a

**Figure 5:**
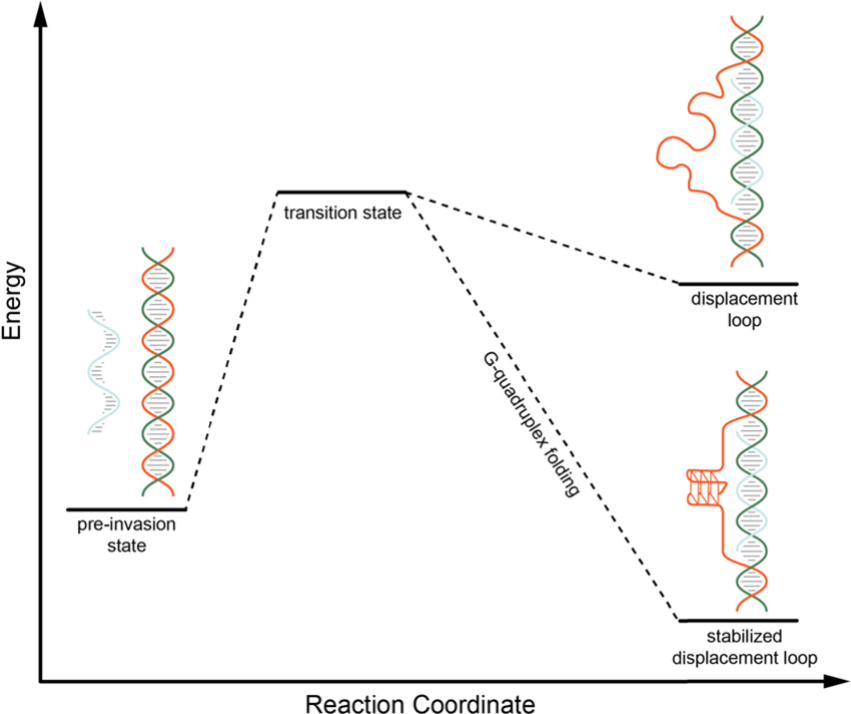
Model of telomeric displacement loop stabilization by G-quadruplex folding within the displaced strand. Under GQ forming conditions, the displaced G-rich strand of the TDL can fold into a GQ stabilizing the overall structure.

TDL upon invasion of the G-rich ssDNA tail permits the G-rich displaced strand to fold into a GQ structure (Figures 5 and S7). Our finding that the kinetics of strand invasion are similar with the G-rich and C-rich invading strands suggests that GQ formation is a late step in the formation of a TDL, serving to thermodynamically stabilize the structure but not accelerate the invasion process.

While it is well documented that single-stranded telomere DNA substrates fold into GQ structures in vitro,^21,45−48^ the prevalence of this structure at telomeres and elsewhere within the genome has been the subject of debate.^23,49^ Interestingly, a recently reported magnetic tweezers study of the promoter region of the c-kit oncogene demonstrated that negative superhelical strain can also drive the B-form to GQ structural transition.^28^ The results of our mechanical analysis of TDLs suggest that the process of strand invasion at telomere DNA targets may provide an opportunity for GQ structures to fold in vivo, as has recently been reported by live cell imaging.^50,51^

## Conclusions

The system we describe in the present study provides a powerful experimental platform for future studies of strand invasion at telomere DNA targets. For example, single-molecule studies using this system can be designed to understand the molecular mechanisms of telomere-associated proteins and enzymes known to resolve D-loop and GQ structures.^52−54^ Moreover, our novel system can be employed to directly study the mechanism of GQ-binding compounds and their possible role in stabilizing TDLs.^55^ Lastly, recent studies have shown that telomeres, long thought to be transcriptionally silent, are transcribed to generate long noncoding telomere repeat-containing RNA (TERRA).^56^ TERRA is implicated in regulating various aspects of telomere biology and is proposed to do so through the formation of RNA loops (R-loops) at telomeres.^57^ Thus, future work utilizing our novel MT-based assay will also focus on the mechanical properties of telomeric R-loops and the molecular mechanism of TERRA-mediated regulation of telomere function.

## Supporting Information Available

Detailed materials and methods and Figures S1–S7 (PDF)
